# Management of Geriatric Ankle Fractures

**DOI:** 10.1007/s11914-025-00940-3

**Published:** 2025-10-27

**Authors:** Lyndon Mason, Chijioke Orji, Jan Szatkowski

**Affiliations:** 1 Liverpool Orthopaedic Trauma Service, Liverpool University Hospital NHS Foundation Trust, Liverpool, UK; 2 University of Liverpool, Liverpool, UK; 3 Indiana University School of Medicine, IU Health, Indianapolis, IN United States of America

**Keywords:** Ankle fracture, Elderly, Osteoporosis, Geriatric, Fixation

## Abstract

**Purpose of Review:**

This review evaluates current management options for managing ankle fractures in geriatric patients with osteoporosis. It addresses the challenges posed by compromised bone integrity and examines operative and non-operative approaches to promote early mobility and functional recovery in elderly individuals.

**Recent Findings:**

Recent studies show that both surgical and non-surgical management can yield comparable functional outcomes, but complication profiles differ. Studies have assessed advanced fixation options such as standard and extended ORIF, hindfoot nailing, and fibular nailing, with growing interest in methods that accommodate poor bone quality and enable early weightbearing. Individualised care pathways and the need for standardisation in rehabilitation protocols are important.

**Summary:**

Personalised and multidisciplinary treatment is essential for this group of patients. No single intervention is optimal; treatment must consider patient frailty, comorbidities, and functional demands. Future research should focus on randomised controlled trials to refine surgical indications and rehabilitation strategies, improving outcomes and reducing complications.

## Introduction

Osteoporotic fractures represent a significant and increasingly prevalent injury among elderly individuals, particularly due to the rising incidence of osteoporosis within an ageing population [1]. Osteoporosis primarily affects postmenopausal women and older men, with an estimated risk of 53.2% in women and 20.7% in men over the age of 50 experiencing osteoporotic fractures at some point in their lives [2]. Although less common than hip or vertebral fractures, osteoporotic ankle fractures significantly affect patient morbidity, functional outcomes, and healthcare costs [1, 3]. In Finland, osteoporosis-related ankle fractures increased in incidence considerably over a 20 year period, rising from 57 per 100,000 persons in 1970 to 130 per 100,000 persons in 1994 [4].

Osteoporotic ankle fractures pose unique clinical challenges due to compromised bone integrity, decreased mineral density, and patient frailty, making traditional management strategies less effective [5]. These fractures often result from minimal trauma but can lead to complex fracture patterns, prolonged recovery, and higher complication rates [6]. Therefore, understanding the pathophysiology, making an accurate diagnosis, understanding the injury by utilizing effective classification methods, and employing individualized management plans specific to osteoporosis-related ankle fractures is likely to improve outcomes.

## Pathophysiology

Osteoporotic bone is characterized by reduced bone mineral density (BMD), disrupted microarchitecture, and compromised bone strength, leading to increased fragility and susceptibility to fractures [7]. The trabecular bone of the distal tibia is particularly susceptible to osteopenia due to its high metabolic activity, thereby increasing the risk of fracture with low-energy trauma [8, 9]. In the context of ankle fractures, osteoporosis presents specific challenges, including a tendency for complex and comminuted fracture patterns and difficulties in achieving stable fixation [10]. The altered bone quality complicates surgical fixation, as osteoporotic bone provides poor screw purchase and stability, necessitating specialised fixation strategies [11].

As noted by Lee et al., ankle fractures in patients older than 50 years of age showed lower bone attenuation on CT in the talus, medial malleolus, lateral malleolus and distal tibial metaphysis as compared to their younger counterparts, especially in females [12]. Gender plays a key role, as women are at a higher risk due to lower peak bone mass than men [13]. Hjelle et al. noted a further association with higher body mass index, especially more complex injuries with syndesmosis instability, although they questioned the association with osteoporosis alone [14]. Similarly, Lee et al. showed no difference in bone mineral density between an ankle fracture group and a normal population, but did note a significantly higher body mass index [15].

Comorbidities like rheumatoid arthritis, type 2 diabetes, and chronic kidney disease impair bone quality and healing potential, increasing the risk of osteoporotic fractures. Genetic predisposition and family history of osteoporosis or fractures further heighten this risk. Other intrinsic factors include poor proprioception and reduced muscle strength, which increase the likelihood of falls, especially in older adults [16]. Extrinsic factors, such as low-energy trauma like slips, trips, or minor impacts, and unsafe environments like uneven surfaces, poor lighting, and lack of supportive footwear, contribute to ankle fractures in individuals with osteoporosis [17]. Smoking accelerates bone loss by reducing osteoblast activity and impairing calcium absorption, whereas excessive alcohol consumption interferes with bone remodelling and balance [18]. Active efforts to mitigate these risks, such as weight-bearing exercises, adequate nutrition, and fall-proof living spaces, are important preventive measures.

## Diagnosis

### Imaging Techniques

Accurate diagnosis of osteoporotic ankle fractures require imaging modalities to evaluate fracture patterns, bone quality, and complications [7]. These techniques assess injury severity and guide treatment strategies.

Radiographs:

Plain radiographs are the initial imaging modality for suspected ankle fractures. Standard views, including anteroposterior, lateral, and mortise projections, provide a comprehensive overview of the ankle’s bony anatomy [7]. Osteoporotic fractures often show diminished bone quality, increased fragility, and comminution due to compromised structural integrity [19]. Cortical thinning, metaphyseal involvement, trabecular bone impaction or collapse, and reduced bone density are also common [20]. While radiographs provide crucial initial information, they may not detect subtle or complex fractures, necessitating further imaging. Non-weightbearing radiographs cannot always identify instability, and further weightbearing imaging is advocated by the British Orthopaedic Association Standards of Trauma guidelines to ensure stability is assessed [21]. Gravity and stress radiographs significantly overestimate instability and have been replaced by weightbearing views in many practices [22].

Advanced Imaging: Computed Tomography.

Computed tomography (CT) can be useful in evaluating osteoporotic ankle fractures, particularly when there is articular involvement or comminution. This can aid in surgical planning, particularly when advanced fixation strategies are considered. Additionally, CT improves the visualisation of trabecular bone structure, revealing the extent of bone loss and overall tissue quality [12]. Subtle fractures are often detected, making it an important diagnostic tool in uncertain cases. CT imaging can lead to tailored management strategies for osteoporotic patients [23].

## Management

### Non Surgical Treatment

**Fig. 1:**
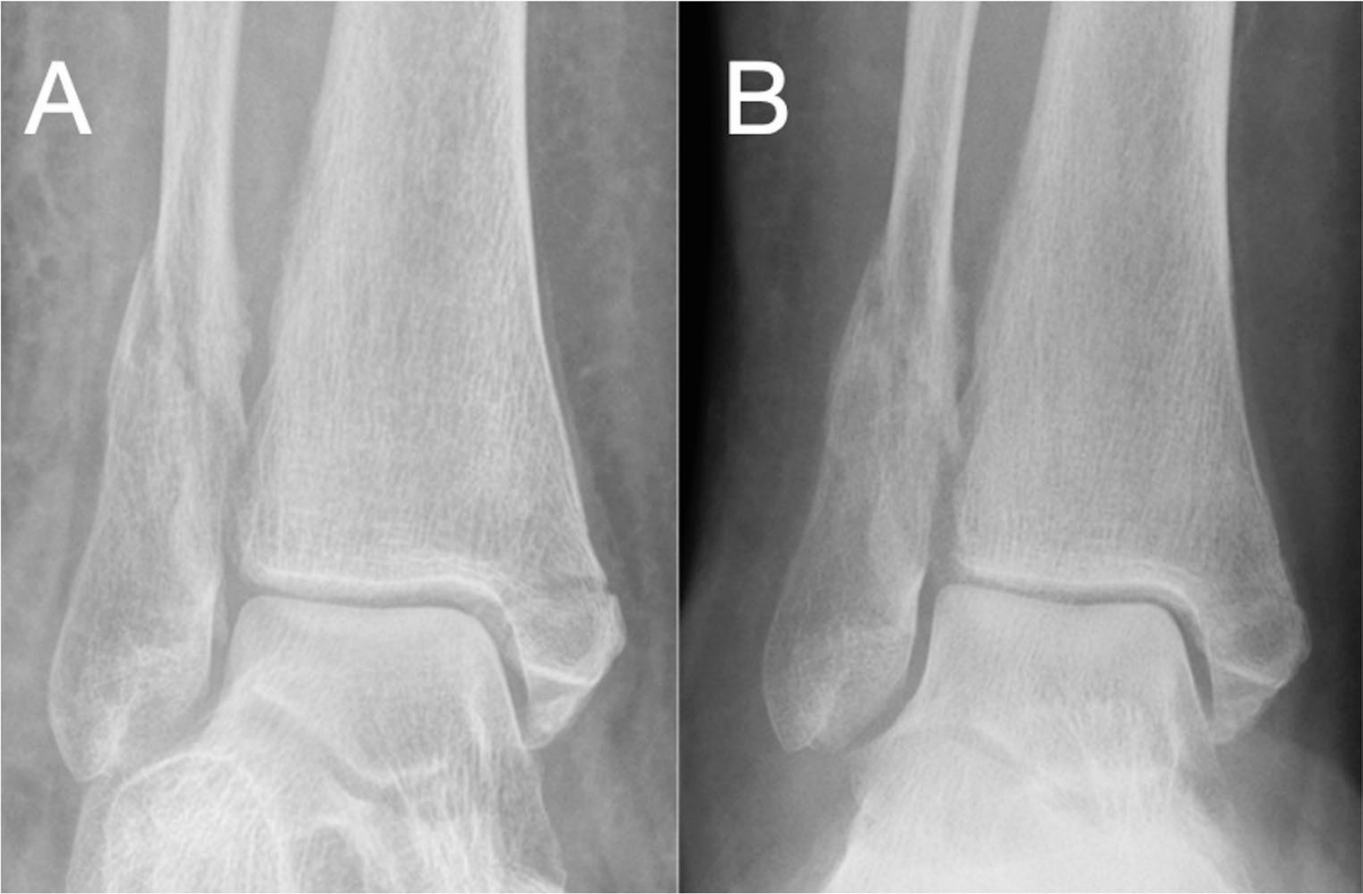
Preoperative (**A**) and 6 weeks postoperative (**B**) radiographs of an unstable ankle fracture treated in a cast

Historically, non-operative management involved immobilisation in casts or braces for minimally displaced fractures or medically unfit patients. Recent insights from the Ankle Injury Management (AIM) trial have shown no difference in functional outcomes at 6 months in patients aged over 60 who were randomized to either close contact casting or surgical fixation (Fig. 1) [24]. Radiographic malunion was more frequent in non-operatively treated fractures, though this did not significantly impact the reported functional outcomes [24]. Further functional analysis was undertaken at 3 years post-treatment. In this follow-up study, only 50.45% (278) were noted to be reduced. However, although malreduction was noted to have significantly worse functional outcomes, this did not reach a minimal clinically important difference [25]. This follow up article to the AIM trial suggested that we should challenge traditional definitions of malunion in geriatric patients, suggesting that radiological findings may not always correlate with functional outcomes. Similar outcomes of lack of correlation between malunion and functional outcomes have been reported in distal radius fractures [26]. Other studies reporting on malunion and functional outcomes in ankle fractures of all age groups have noted marked functional loss with malunion, and thus, the findings by Knight et al. may be limited to the older age group it examined [27].

### Surgical Intervention

A large UK multicentre study was conducted to investigate the epidemiology of ankle fractures complicated by significant comorbidity and patient factors, as well as the use of specialist surgical techniques [28]. Although not limited to elderly patients, it was noted that out of 1360 patients, 90.2% (1227) underwent surgery. Standard open reduction internal fixation (ORIF) was undertaken in 78.9% (1073), extended ORIF in 3.25% [29] and hind foot nail procedure in 8.1% (111). Standard ORIF, however, did not differentiate locking and non-locking plate fixation. The study illustrated the varying methods different centres are undertaking to treat osteoporotic ankle fractures.

**Fig. 2:**
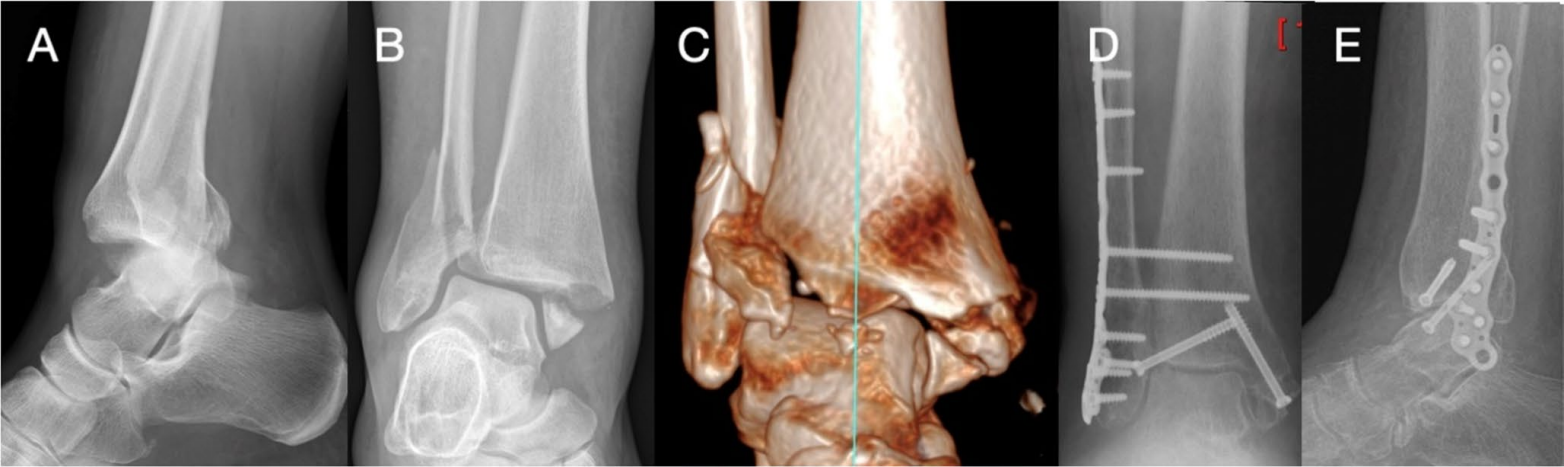
Preoperative lateral (**A**) and anteroposterior (**B**) radiographs and 3D CT reconstruction (**C**) of an unstable ankle. Weight-bearing anteroposterior (**D**) and lateral (**E**) radiographs taken at 3 months post extended ORIF fixation using a locking plate and fibular pro tibia fixation

**Fig. 3:**
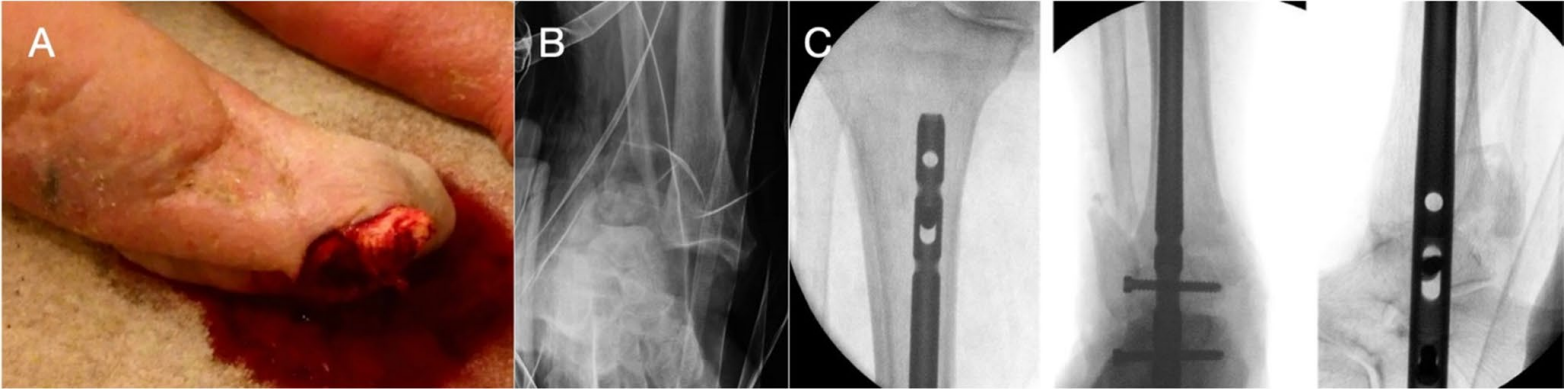
Picture taken by ambulance crew (**A**) at time of injury showing extruding tibia out of medial skin, with foot folded up behind leg. (**B**) shows trauma radiograph of dislocated ankle and (**C**) intraoperative imaging of a retrograde femoral nail (Stryker T2) being used to span the zone of injury. Tibia shortened to allow primary closure of medial skin

**Fig. 4:**
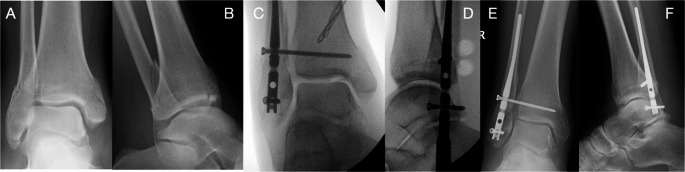
Preoperative anteroposterior (**A**) and lateral (**B**) radiographs and intraoperative radiographs (**C **and **D**) of a fibular nail fixation of an unstable ankle fracture. Weight-bearing anteroposterior (**E**) and lateral (**F**) radiographs taken at 6 months

Studies analyzing specific techniques are limited by small sample sizes and low-level evidence. Standard ORIF is still the most common practice; however, further developments in fixation methods aim to reduce complications and allow early mobilization. Theoretically, this is achieved by increasing the strength of the fixation, reducing the dependence on the patient’s own bone strength and reducing soft tissue insult. Studies reporting on the techniques of extended ORIF (Fig. 2), tibiotalocalcaneal (Fig. 3) nail, and fibular nail (Fig. 4) are reported in Table 1.

**Table 1:** List of publications reporting different techniques for ankle fixation in the elderly

Name of Paper	Method of Fixation	Number	Mean Age	Follow-up (months)	Functional Score	Number of Open Fractures	Complications
Case Series							
Hasan et al. [30]	Antegrade intramedullary tibial nail	15	83	12	NA	6	14 patients returned to baseline activity; no significant complications reported
Appleton et al. [31]	Fibula nail	NA	Elderly	NA	NA	NA	NA
Bugler et al. [32]	Fibular nail	105	64.8	72	OMAS 65	0	7 losses of fixation; 5 wound infections
Ramasamy et al. [33]	Fibular nail	11	67.2	25.9	OMAS good/excellent in 88%	0	1 ankle subluxation requiring fusion
O’Daly et al. [34]	Gallagher Nail	9	81	34	NA	0	No wound complications; all fractures healed satisfactorily
Lemon et al. [35]	Tibiotalarcalcaneal nail	12	84	15.4	OMAS 61	0	No wound complications; one patient died post-operatively
Corin et al. [36]	Tibiotalarcalcaneal nail	70	84	12	NA	45	5 infections, 10 locking bolt removal, 2 below knee amputations
Balziano et al. [37]	Tibiotalarcalcaneal nail	22	80.8	NA	NA	1	1 DVT, 2 wound dehiscence, 1 periprosthetic fracture, 1 cellulitis requiring hardware removal
Al-Nammari et al. [38]	Tibiotalarcalcaneal nail	48	82	6	OMAS 57	19	2 superficial infections, 1 deep infection, 3 screw issues, 2 valgus malunion, 1 amputation
Baker et al. [39]	Tibiotalarcalcaneal nail	16	73	21	NA	NA	No deep infections or wound complications
Taylor et al. [40]	Tibiotalarcalcaneal nail	31	NA	13.6	NA	NA	2 superficial infections, 3 deep infections, 3 screw breakages
Jonas et al. [41]	Tibiotalarcalcaneal nail	31	NA	NA	OMAS 45	0	3 peri-prosthetic fractures; 2 nail failures
Kotsarinis et al. [42]	Tibiotalarcalcaneal nail	32	80.2	12	OMAS 45	NA	2 surgical site infections, 18.8% reintervention rate
Ebaugh et al. [43]	Tibiotalarcalcaneal nail	27	66	29.6	NA	6	18.5% surgical complications, 12% non-union
Armstrong et al. [44]	Tibiotalarcalcaneal nail	21	76	NA	NA	21	6 superficial infections, 1 further soft tissue surgery
Amirfeyz et al. [45]	Tibiotalarcalcaneal nail	13	78.9	11	OMAS 50	0	No significant complications; all patients showed evidence of fracture union
Persigant et al. [46]	Tibiotalarcalcaneal nail	14	79.6	11	Parker score mean 2.7 (48 weeks)	3	1 deep infection, 1 distal screw migration
Comparative studies							
White et al. [47]	Fibular nail, ORIF	100	74	12	OMAS 63 (Nail), 61 (ORIF)	0	Significantly fewer wound infections in nail group
Keene et al. [24]	Close Contact Casting (CCC), ORIF	620	60+	6	OMAS 64.5 (CCC), 66 (ORIF)	0	More malunion and non-union with CCC; higher infections and reoperation in ORIF
Eyre-Brook et al. [48]	Extended ORIF, Tibiotalarcalcaneal nail	68	60+	NA	NA	NA	Higher revision rates in IMN group; non-union and delayed wound healing higher in IMN
	ORIF, Extended ORIF, Tibiotalarcalcaneal nail	1360	53.1	7.8	NA	275	Wound infection/breakdown (ORIF 10.7%, HFN 11.7%), VTE events (ORIF 0.8%, HFN 2.7%), diabetic complications higher (15.8%)
Georgiannos et al. [49]	ORIF, Tibiotalarcalcaneal nail	87	70+	14	OMAS 56.9 (TTC), 56.6 (ORIF)	0	3 in TTC group, 12 in ORIF group
Fourman et al. [50]	ORIF, Tibiotalarcalcaneal nail, External Fixation, Amputation	113	75.2	NA	NA	113	High rate of unplanned OR (esp. external fixation)

### Extended ORIF Fixation

Extended ORIF is usually described as fixation with locked screws, spanning through the fibula into the tibia. Also called fibula pro-tibia (FPT) fixation, it improves rotational stability and distributes forces effectively through the stronger tibial cortex [51]. Okoro et al. found in a biomechanical study that the use of fibula pro-tibia fixation demonstrated approximately 3 times the level of torque achieved at 30 degrees external rotation and twice the failure torque in comparison to standard locking plate fixation [52]. They proposed the 3, 3, 3 rule for use of this adjunctive technique; Fixation with 3 screws across 3 cortices starting 3 cm above the tibial plafond using locking plate technology [52].

In the Hindfoot Ankle Reconstruction Nail Trial (HARnT), comparison of extended ORIF (including FPT) with standard ORIF by propensity matching for high-risk individuals showed that extended ORIF had a significantly better complication profile than standard ORIF [28]. Further comparative analysis by Eyre-brook et al. between FPT fixation and hindfoot nailing (HFN) in unstable ankle fractures in patients > 60 years showed a higher complication rate in the HFN group [48]. These included increased rates of wound complications, non-union and longer hospital stays. Extended ORIF also resulted in a significantly lower surgical revision rate, which is consistent with the findings of Said et al., who applied the technique to tibial nonunions with concurrent fibula fractures, demonstrating union and no adverse effect on ankle range of motion [53]. Despite promising outcomes, the evidence supporting extended ORIF remains weak due to lack of RCTs, as most of the current evidence is from retrospective reviews and case series [48, 54].

### Hindfoot Nail

In a single-centre randomised control trial comparing standard ORIF with non-locking plates vs. HFN in the elderly, Georgiannos et al. found a significantly reduced length of stay, fewer complications and no significant difference between post-operative Olerud-Molander Ankle Score (OMAS) score with HFN [49]. These findings are consistent with the results of the meta-analysis conducted by Tan et al. [55], which revealed an 81.5% rate of return to pre-injury mobility aid utilisation and a comparatively low incidence of complications, thereby reinforcing the role of HFN in elderly patients with low demand. Lemon et al. recommended removing the implant after the fracture had healed [35]. However, 50% of their patients refused removal of nail since they felt more stable with the nail in place than they did before injury. This patient preference for nail retention highlights the functional reassurance it may offer.

In the HARnT study, comparison was made of hindfoot nails undertaken with and without fusion. It was found that the removal of metalwork was more common in the fusion group, and a trend toward an increase in wound complications compared to no fusion [28]. This suggests that fusion may not add any clinical benefit to patients, although the method of fusion was not described in the study and the numbers were small.

### Fibula Nail

Fibular nail use in the elderly has been increasingly advocated due to its reduction in soft tissue complications post-surgery [47]. Nevertheless, it is not without its problems with the HARnT study reporting in 42 patients who had a fibular nail, a total of 10 patients (28.9%) required further surgery, 3 (7.1%) for failed construct and 4 (9.5%) for removal of metalwork [28]. White et al., in a randomised trial between fibular nail and standard ORIF, showed equivalent functional outcomes (OMAS 63 *versus* 61, *p* = 0.61) with a significantly lower rate of wound infections in the fibular nail group [47]. It should be noted, however, the disparity between the seniority of surgeons in the groups analysed, creating a selection bias. Comparative evidence from a meta-analysis supports the clinical advantages of fibular nail over standard ORIF due to the lower rate of wound complications and lower infection rate, although again, there was no difference in functional scores that met minimal clinically important difference [56]. In another meta-analysis, they concluded that there was insufficient evidence for changing practice from plating of unstable distal fibular fractures to intramedullary fixation [57]. 

### Rehabilitation

In the UK, the national guidance published by the British Orthopaedic Association Standards states that “all surgery in the frail patient should be performed to allow full weightbearing for activities required for daily living, within 36 hours of admission” [58]. Most technique papers allow immediate weight bearing except the AIM trial, which restricted weight bearing for 4 weeks when casted, and many of the fibular nail papers [24, 31, 32, 47]. The Fragility Fracture Post Operative Mobilization (FFPOM) multicenter audit in the UK analysed 19,557 elderly patients, showing a significant disparity in weightbearing restrictions despite this national guidance [29]. Early post operative mobilization is critical in obtaining satisfactory outcomes, however, Fourman et al. noted that pre-operative mobility was also one of the most predictive factors in occurrence of complications in 90-days and 1 year mortality [50].

## Conclusion

Osteoporotic ankle fractures require nuanced management due to compromised bone quality, increased complexity, and risk of complications in the ageing population. No single operative technique is universally superior; patient-specific factors, such as comorbidities, mobility, and bone quality, should guide the surgical management. The aims of treatment should be to allow immediate weight-bearing activity. These principles are especially relevant in osteoporotic patients, where prolonged immobilisation worsens bone loss and functional decline.

## Data Availability

No datasets were generated or analysed during the current study.
